# Myringosclerosis in patients with chronic renal failure: comparative analysis with a control group

**DOI:** 10.1016/S1808-8694(15)30594-2

**Published:** 2015-10-18

**Authors:** Silvio Caldas Neto, Fábio José Delgado Lessa, Gabriel Alves, Nelson Caldas, Mariana de Carvalho Leal Gouveia

**Affiliations:** 1 Habilitation degree (livre docência), Adjunct Professor and Coordinator of the Otorhinolaryngology Discipline, Pernambuco Federal University (UFPE).; 2 Master degree in Public Health. Assistant Professor of the Speech Therapy Basics Discipline, UFPE.; 3 Physician, otorhinolaryngology specialist.; 4 Assistant Professor, UFPE.; 5 Doctoral degree, physician, voluntary tutor at the Otorhinolaryngology Unit, Clinical Hospital - UFPE. This study was done in the Otorhinolaryngology Unit and the Nephrology Unit of the Clinical Hospital, - UFPE.

**Keywords:** kidney failure, middle ear, otoscopy

## Abstract

Myringosclerosis is a scar of the tympanic membrane lamina propria, resulting from trauma or inflammation, characterized by proliferation of collagen, hyalinization, calcium and phosphate deposits and cartilaginous or osseous metaplasia of the middle ear mucosa, a sequence that is similar to that taking place in other types of pathologic calcification, common in chronic renal failure (CRF).

**Aim:**

To verify the influence of chronic renal failure on the prevalence of myringosclerosis.

**Method:**

The otoscopic examination was done in 341 chronic renal failure patients and in 356 normal control individuals. The frequency of positive otoscopies was compared between the two groups, based on individual variables and those pertaining to CRF.

**Results:**

11.7% of the patients had a positive otoscopy in the CRF group, compared to 5.1% in the control group. There was no statistical diference in the frequency of myringosclerosis acording to gender, ethnic group, time of dialysis or serum minerals. The groups had a wide age range.

**Conclusion:**

Although the findings of this study suggest a higher ocurrence of myringosclerosis in patients with renal disease, they do not provide a basis for stating that there is any relation between renal failure and tympanic alterations.

## INTRODUCTION

Tympanosclerosis is histologically characterized by hyaline degeneration of the middle ear mucosa, followed by calcium and phosphorus deposits in the submucosa,^1^, ^2^ that becomes hardened, even bone-like.

This condition may occur in the tympanic membrane, where it is named myringosclerosis,^3^ easily identified during otoscopy; it may also occur in other middle ear sites, with a preference for the attic.

Other forms of pathological calcification found in different bodily tissues are similar to tympanosclerosis in that they involve matrix vesicles, fibroblasts, macrophages and various enzymes.^4^, ^5^, ^6^, ^7^, ^8^, ^9^, ^10^, ^11^, ^12^, ^13^ These forms of calcifications are the result of calcium and phosphorus dysmetabolism, and are found in other systemic conditions, including chronic renal failure (CRF). Based on this, our study aimed to find a possible cause-effect relation between CRF and tympanosclerosis.

## OBJECTIVE

The aim was to assess the influence of CRF on the occurrence of tympanosclerosis.

## SERIES AND METHOD

This paper is a cross-sectional epidemiological trial with a study group (group A) and a control group (group B). Group A was composed of 341 CRF patients in hemodialysis therapy, and group B was composed of 356 caretakers of patients from the outpatient and infirmary sectors of the Otorhinolaryngology Unit with no otological complaints and no kidney disease. The 341 study group patients were recruited from three separate hemodialysis units in the city of Recife.

Age ranged from 10 to 88 years; the mean age in group A was 46.5 years (SD ± 14.84), and the mean age in group B was 39.9 years (SD ± 14.39). There were 422 females and 275 males in the total sample, of which 101 were white, 101 were black and 495 were brown.

Data collection was done between March and June 2002. Subjects underwent a questionnaire (Annex A) and an otoscopic exam.

The closed-question questionnaire was applied in an interview and contained identification data (sex, age, color) and the otological morbidity history (history of otalgia and/or otorrhea).

Otoscopy was done with a Welch Allyn model 71000-C 3.5 V rechargeable battery (72300) otoscope, always by two examiners who remained the same for all of the subjects.

Upon inclusion, serum calcium, phosphorus and parathormone (PTH) were investigated in 225 patients from one of the hemodialysis clinics.

Otoscopy was positive if a typical plaque was seen on the tympanic membrane. In elderly patients, naturally occurring (for this age) diffuse opacification of the tympanic membrane was not considered myringosclerosis.

Normal serum calcium was within 8.4 and 10.2 mg/dl. Normal serum phosphorus was between 2.7 and 4.5mg/dl. Normal PTH levels were between 14 and 106 pg/ml.

A positive history of otitis was defined as past episodes of otalgia or otorrhea.

The duration of dialysis was counted from the first recorded session in the patient file until the day in which otoscopy was done.

The Epi-Info 6.0 StatCalc module formula for calculating “n” was used at a 5% significance level and an 80% power. Data analysis was done using descriptive statistics, using central trend and dispersion measurements, and analytical statistics, using the Yates corrected chi-square test (x2) (p<0.05).

## RESULTS

The prevalence of myringosclerosis in 697 patients was 8.3% (n = 58). The prevalence in group B was 5.1% (n = 18); the prevalence in CRF patients was 11.7% (n = 40). The latter group had a 2.5 times chance of having a positive otoscopy compared to the control group (CI = 1.21 - 1.77; PR = 1.46; p = 0.002), as shown in Table 1.

**Table 1:** Frequency of myringosclerosis in group A and B subjects.

	myringosclerosis	no myringosclerosis	TOTAL
	n%	n%	
GROUP A	40 11,7	301 88,3	341 100
GROUP B	18 5,1	338 94,9	356 100

TOTAL	58 8,3	639 91,7	697 100

PR = 1.46; CI = 1.21- 1.77; p = 0.002

There were other eventual otoscopic findings, such as tympanic perforation, in six group A subjects and external otitis in two group A subjects.

### Myringosclerosis and a history of otitis

Of 58 subjects with myringosclerosis, 10 (17.2%) had a history that suggested middle ear inflammatory disease, while 114 (17.8%) of 639 subjects with no myringosclerosis reported prior episode(s) of otologic disease, which was not statistically significant (p = 0.94). Groups A and B had a similar otological history of disease, which was positive in 64 (18.7%) of the CRF patients and in 60 (16.9%) of the control group subjects (PR = 1.07; CI = 0.88 - 1.29; p = 0.57), and which was not statistically significant, as shown in Table 2.

**Table 2:** Frequency distribution of past otitis in group A and B subjects.

	past otitis	no past otitis	TOTAL
	n%	n%	n%
GROUP A	64 18,7	277 81,3	341 100
GROUP B	60 16,9	296 83,1	356 100

TOTAL	124 17,8	573 82,2	697 100

PR = 1.07; CI = 0.88 - 1.29; p = 0.57

On the other hand, if we exclude from both groups those subjects that had a history of otitis (64 in group A and 60 in group B), we are left with 573 subjects, 277 in group A and 296 in group B. Among the 277 group A patients, 34 (12.3%) had myringosclerosis; among the 296 control group subjects, 14 (4.7%) had myringosclerosis (Table 3). These findings are even more statistically significant compared to the original sample that included all of the patients (PR = 1.53; CI = 1.25 - 1,88; p = 0.001).

**Table 3:** Frequency of myringosclerosis in groups A and B with no history of otitis.

	myringosclerosis	no myringosclerosis	TOTAL
	n%	n%	
GROUP A	34 12,3	243 87,7	277 100
GROUP B	14 4,7	282 95,3	296 100

TOTAL	48 8,4	525 91,6	573 100

PR = 1.53; CI = 1.25 - 1.88; p = 0.001

### Myringosclerosis and gender

There were 422 (60.5%) female subjects and 275 (39.5%) male subjects. Among the subjects that presented myringosclerosis, 35 (60.3%) were female and 23 (39.7%) were male (p = 0.91).

In the CRF group there were 153 (44.9%) males and 188 (55.1%) females. In the control group, there were 87 (24.5%) males and 269 (75.5%) females (x2 = 67.41; p < 0.001), as shown in Table 4.

**Table 4:** Distribution of subjects according to sex in groups A e B.

	male	female	TOTAL
	n%	n%	n%
GROUP A	153 44,9	188 55,1	341 100
GROUP B	87 24,5	269 75,5	356 100

TOTAL	240 34,4	457 65,6	697 100

x^2^ = 67.41; p < 0.001

### Myringosclerosis and color

In the full sample there were 101 white subjects (14.5%), 101 black subjects (14.5%) and 495 brown subjects (71%).

Table 5 shows that in group A these numbers were 41 white (12.0%), 60 black (17.6%) and 240 brown (70.4%); in group B these numbers were 60 white (16.9%), 41 black (11.5%) and 255 brown (71.6%). The groups were, therefore, similar in skin color (x^2^= 7.28; p = 0.02).

**Table 5:** Distribution of subjects according to color in groups A e B.

	white	black	brown	TOTAL
	n%	n%	n%	n%
GROUP A	41 12,0	60 17,6	240 70,4	341 100
GROUP B	60 16,9	41 11,5	255 71,6	356 100

TOTAL	101 14,5	101 14,5	495 71,0	697 100

x^2^ = 7,28 p = 0,02

Among the subjects with positive otoscopies for myringosclerosis, 11 (19.0%) were white, 10 (17.2%) were black and 37 (63.8%) were brown. In subjects with no myringosclerosis 90 (14.1%) were white, 91 (14.2%) were black and 458 (71.7%) were brown (x2 = 1.67; p = 0.43).

### Myringosclerosis and age

The sample was grouped into four age ranges (≤20 years, 21 to 40, 41 to 60, and >60 years). Of 697 subjects, 29 (4.2%) were aged 20 years or less; 297 (42.6%) were aged between 21 and 40 years; 275 (39.4%) were aged between 41 and 60 years, and 96 (13.8%) were aged over 60 years (Chart 1).

**Graph 1:**
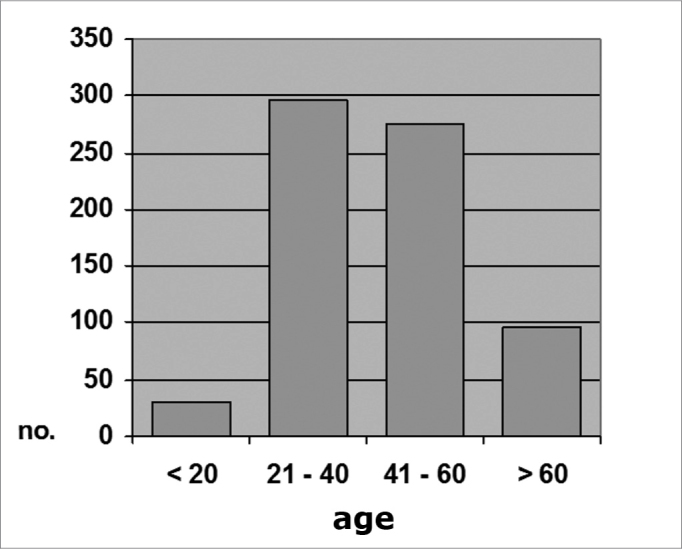
Distribution by age range, All individuals (N=697)

In the CRF group, 8 patients (2.3%) were aged 20 years or less; 118 (34.6%) were aged between 21 an 40 years; 150 (43%) were aged between 41 and 60 years; and 65 (19.1%) were aged over 60 years (Chart 2). The mean age in this group was 46.5 years (SD ± 14.84), ranging from 16 to 88 years.

**Graph 2:**
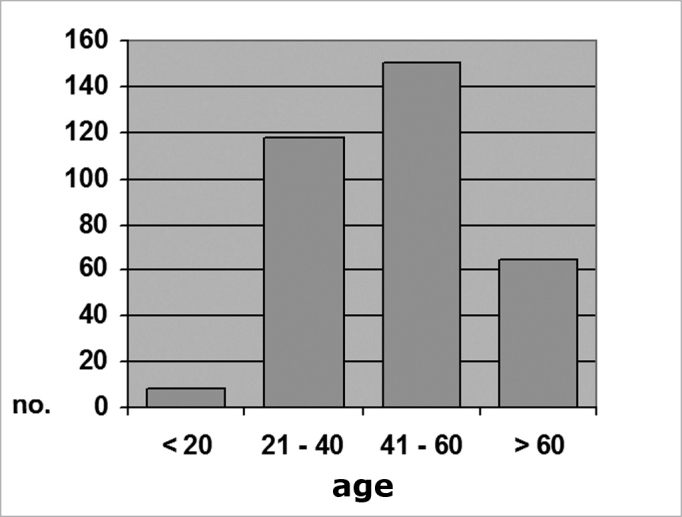
Distribution by age range, Group A (N = 341)

In the control group, 21 subjects (5.9%) were aged 20 years or less; 179 (50.3%) were aged between 21 an 40 years; 125 (35.1%) were aged between 41 and 60 years; and 31 subjects (8.7%) were aged over 60 years (Chart 3). The mean age in this group was 39.9 years (SD ± 14.39), ranging from 10 to 82 years.

**Graph 3:**
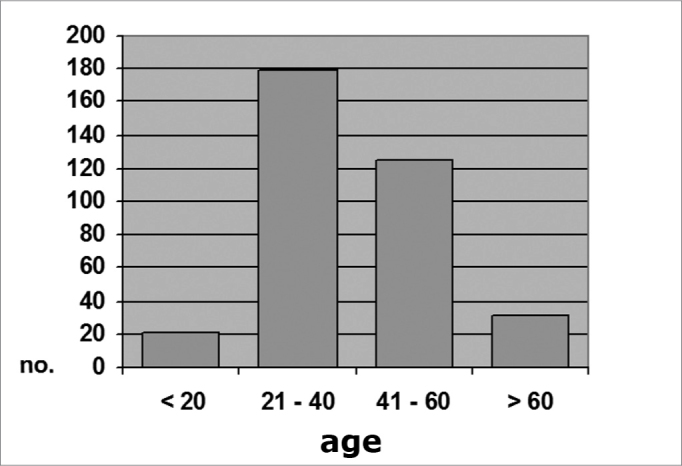
Distribution by age range, Group B (N = 356)

Looking at the most prevalent age groups (21 to 40 years, and 41 to 60 years), the following results are seen (Tables 6 and 7): among 188 group A patients aged between 21 and 40 years, 12 (10.1%) presented myringosclerosis; among 179 control group subjects in the same age bracket, 12 (6.7%) presented myringosclerosis (p = 0.39). In the 41 to 60 years bracket, myringosclerosis was found in 16 (10.7%) of 150 group A patients, and in six (4.8%) of 125 group B subjects (p = 0.11).

**Table 6:** Frequency of myringosclerosis in group A and B subjects aged between 21 and 40 years.

	myringosclerosis	no myringosclerosis	TOTAL
	n%	n%	n%
GROUP A	12 10,1	106 89,9	118 100
GROUP B	12 6,7	167 93,3	179 100

PR = 1,29 CI = 0,84 -1,97x^2^ = 0,73 p = 0,39

**Table 7:** Frequency of myringosclerosis in group A and B subjects aged between 41 and 60 years.

	myringosclerosis	no myringosclerosis	TOTAL
	n%	n%	n%
GROUP A	16 10,7	134 89,3	150 100
GROUP B	6 4,8	119 95,2	125 100

PR = 1,37 CI = 1,04 -1,82x^2^ = 2,44 p = 0,11

### Myringosclerosis and duration of dialysis

In the full sample (with and with no myringosclerosis), 50.4% had been on dialysis for less than 2 years, 25.3% had been on dialysis for between 2 and 5 years, and 24.3% had been on dialysis for over 5 years.

Among CRF patients with otoscopically diagnosed myringosclerosis, 16 (40%) had been on dialysis for less than 2 years, 12 (30%) had been on dialysis for between 2 and 5 years, and 12 (30%) had been on dialysis for over 5 years. Among CRF patients with no myringosclerosis, 156 (42.4%) had been on dialysis for less than 2 years, 74 (24.6%) had been on dialysis for between 2 and 5 years, and 71 (23.6%) had been on dialysis for over 5 years (x^2^ = 1.99; p = 0.37). These numbers are shown on Charts 4 and 5.

### Myringosclerosis and serum calcium

Serum calcium was measured in 208 of 341 group A patients. Of these, hypocalcemia was present in four patients (2%), normal calcium levels were normal in 96 patients (46%), and hypercalcemia was present in 108 patients (52%).

Table 8 shows that only one of four hypercalcemic patients presented myringosclerosis. Otoscopy revealed a myringosclerotic plaque in 11 (11.5%) of 96 normal calcium level patients; the remaining 85 (88.5%) of these 96 patients had no myringosclerosis. Myringosclerosis was found in 14 (13%) of 108 hypercalcemic patients, and the remaining 94 (87%) of these 108 patients had normal tympanic membranes (x^2^ = 0.69; p = 0.70).

**Table 8:** Frequency of myringosclerosis in group A patients presenting low, normal and elevated serum calcium levels.

	Hypocalcemia	Normocalcemia	Hypercalcemia
	n%	n%	n%
myringosclerosis	1 25	11 11,5	14 8,4
no myringosclerosis	3 75	85 88,5	94 91,6

TOTAL	4 100	96 100	108 100

x^2^ = 0,69 p = 0,70

**Graph 4:**
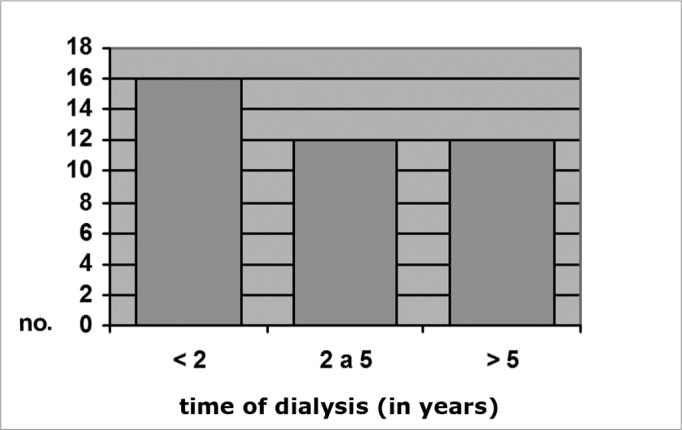
Distribution by time of dialysis, Group A c/ MS (N = 40) Dialysis time (in years)

**Graph 5:**
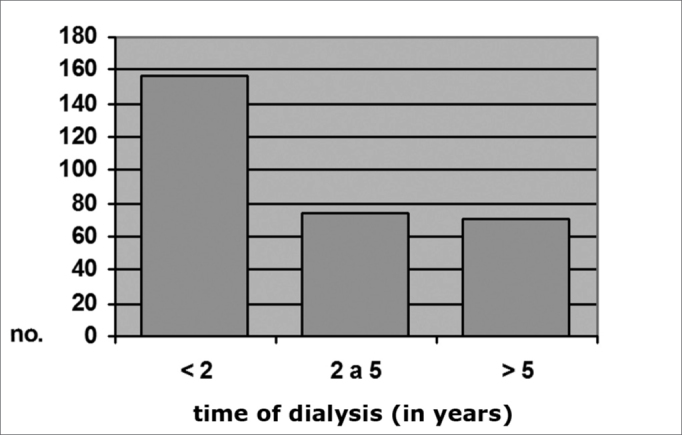
Distribution by time of dialysis, Group A s/ MS (N = 301)

The lowest calcium level in these patients was 4.2mg/dl and the highest calcium level was 108.0mg/dl (SD ±12.84mg/dl).

### Myringosclerosis and serum phosphorous

Serum phosphorus was measured in 206 group A patients. Low phosphorus levels were found in 16 (7.8%) of these patients, while 68 patients (33%) had normal phosphorus levels, and 122 patients (59.2%) presented elevated phosphorus levels.

Table 9 shows the frequency of myringosclerosis in each subgroup (low phosphorus levels: 12.5% with myringosclerosis; normal phosphorus levels: 13.2% with myringosclerosis; elevated phosphorus levels: 12.3% with myringosclerosis), x^2^ = 0.04 and p = 0.982.

**Table 9:** Frequency of myringosclerosis in group A patients presenting low, normal and elevated serum phosphorus levels.

	Hipophosphatemia	Normophosphatemia	Hyperphosphatemia
	n%	n%	n%
myringosclerosis	2 12,5	9 13,2	15 12,3
no myringosclerosis	14 87,5	59 86,8	107 87,7

TOTAL	16 100	68 100	122 100

x^2^ = 0,04 p = 0,982

The mean phosphorus level was 5.92mg/dl; the minimum level was 2.20mg/dl and the maximum level was 62.0mg/dl (SD ± 5.26mg/dl).

### Myringosclerosis and serum PTH

Serum PTH was measured in 158 CRF patients, of which 46 (29%) had below normal PTH, 44 (28%) had normal serum PTH, and 68 (43%) had elevated serum PTH.

Myringosclerotic plaques were found in 5 (10.8%) of 46 low-PTH patients, in 10 (22.7%) of 44 normal PTH patients and in 8 (11.7%) of 68 elevated PTH patients (x^2^ = 3.29; p = 0.192); these results are presented in Table 10.

**Table 10:** Frequency of myringosclerosis in group A patients presenting low, normal and elevated PTH.

	Low pth	Normal pth	Elevated pth
	n%	n%	n%
myringosclerosis	5 10,8	10 22,7	8 11,7
no myringosclerosis	41 89,2	34 77,3	60 88,3

TOTAL	46 100	44 100	68 100

x^2^: = 3,29 p = 0,192

The mean serum PTH level was 285.3pg/ml. Values ranged from 1pg/ml to 1,923pg/ml (SD ± 380.65pg/ml).

## DISCUSSION

Tympanosclerosis is the end stage of a process that starts as acute or chronic inflammation of the middle ear lamina propria.14 Many authors have described the pathology that leads to tympanosclerosis; this process begins with excessive collagen production in the lamina propria of the middle ear mucosa, followed by hyalinization, calcification and bone or cartilage metaplasia.^1^, ^5^, ^11^, ^15^, ^16^

The etiology and pathogeny, and the reasons why only some patients develop this type of process, are not fully understood. A few studies have attempted to establish an etiological and pathogenic association with certain factors; the papers published by Schiff et al.,^17^ and Schiff & Yoo,^18^ describe a relation between tympanosclerosis and the autoimmune reaction, and by Koç & Uneri,^19^ have raised the hypothesis that there might be a genetic predisposition.

The tympanosclerotic plaque calcification process is similar to other types of pathological calcification. Ectopic calcification has long been studied both structurally and ultra-structurally. There are basically two methods for depositing calcium crystals in tissues other than bone, namely metastatic and dystrophic calcification. Metastatic calcification occurs passively by serum calcium phosphate crystal precipitation in patients with certain diseases in which serum levels of these crystals is elevated.^20^ This type of calcification appears to depend on hypercalcemia and hyperphosphatemia, which have an essential role in the process.^21^ Dystrophic calcification involve a complex active cell mechanism that has been widely researched without much success. It is seen as calcification of arteries and viscera in chronic renal disease.^20^ Certain authors have questioned the role of elevated serum calcium and phosphorus in the pathophysiology of this type of calcification,^20^ although various papers have associated these two conditions.^22^, ^23^, ^24^

An altered glomerular filtration rate due to kidney disease leads to phosphate retention in the serum. Excess serum phosphorus tends to bind to free calcium to form precipitates that may originate ectopic calcification. Precipitation in turn reduces serum calcium levels, which stimulates the parathyroid glands to produce PTH; this hormone acts on bone metabolism, and may demineralize bone; the minerals that are released reach the bloodstream and may initiate new calcification.^20^, ^25^, ^26^ There seems to be a failure in calcium and parathyroid feedback mechanisms, so that this type of dysmetabolism is perpetuated. In this situation, PTH secretion is not adequately inhibited; serum PTH levels may actually be elevated due to decreased calcitriol levels.^25^

It seems almost certain that calcium and phosphorus dysmetabolism, which is present in chronic renal disease due to the condition itself or due to dialysis, is the basis of ectopic calcification, particularly in the cardiovascular system and viscera. Rather than a single mechanism, it appears that there are various etiologic and pathogenic causes underlying different types of calcification, which explains the biochemical variations that are found in these patients.

Matrix vesicles were described in 1969 as essential components of pathological calcification in various tissues.^12^, ^13^ These vesicles are found within the collagen matrix in connective tissue; electron microscopy has revealed its mineral content. Such vesicles originate from the cell membrane of cells that have undergone apoptosis, and diffuse into the collagen matrix; they eventually coalesce and form a calcium phosphate plaque.^12^, ^13^, ^22^ This process has been demonstrated in atheromatous plaques in CRF patients; there is strong evidence suggesting that hypercalcemia and hyperphosphatemia participate in the origin of these vesicles.^22^, ^27^ Matrix vesicles also participate in other types of calcification, including tympanosclerosis.^4^

Other cellular and hormonal elements have been found to participate actively in tympanosclerotic calcification and in the calcification of other tissues. Macrophages, for instance, may become osteoclasts and produce osteoactive proteins.^6^, ^7^, ^11^ Alkaline phosphatase^28^ and osteopontine, a bone matrix protein found in various calcified tissues,^7^, ^8^, ^9^, ^10^ may also participate in this process. It is clear that there are significant similarities between tympanosclerotic calcification and that which involves other tissues in CRF patients or those with other systemic diseases.

Such similarities have led to the hypothesis that tympanosclerosis, which is initiated by local inflammation following trauma or acute and chronic infection of the middle ear mucous membrane, might arise regardless of any association with other diseases. On the other hand tympanosclerosis could be influenced by the typical dysmetabolism of CRF patients, in which the prevalence of tympanosclerosis would be increased.

Groups A and B were similar relative to the history of otitis and skin color, but differed in relation to age and sex.

Table 2 shows data suggesting that there was a statistically significant difference between the occurrence of myringosclerosis in CRF patients and in the control group (11.7% versus 5.1%). Group A patients had a 1.46 higher risk of developing myringosclerosis compared to healthy subjects. These numbers demonstrate a clear association between CRF and myringosclerosis, and may also be used to roughly estimate the prevalence of myringosclerosis in our population (5.1%), a datum that is not found in the literature we surveyed.

Table 2 reveals that both groups were similar relative to their history of otitis; this variable, therefore, does not affect the results shown on Table 1. Still, to exclude any possibility that this influence might be present, cases with no history of otitis in both groups were also analyzed. Although the samples were reduced by using this exclusion criterion, it became clear that the statistical significance relative to the occurrence of myringosclerosis was further increased in favor of group A (12.3% versus 4.7%), where the risk was 1.53 higher. A memory bias should also be taken into account, as many of these patients were elderly persons, and might not have remembered their clinical past precisely; this bias, however, was present in both groups.

There are no published papers that unequivocally attribute an increased predisposition for tympanosclerosis to one sex over the other. Some papers that have reported sex prevalence have shown that there is no sex-specific difference;^2^, ^29^, ^30^ two papers found a higher prevalence in males.^31^, ^32^ These two studies, however, admit that the increased male prevalence was due to the well-known higher incidence of middle ear inflammatory disease in men.

The sample groups in this study were different relative to sex (p < 0,001); there were many more females in group B. This might be explained by the fact that caretakers of the otorhinolaryngology outpatient and infirmary patients formed this group; these persons are socially and economically similar to the patients enrolled in the study, but are mostly female. An analysis of the occurrence of myringosclerosis and sex in groups A and B) reveals that, although there is no statistically significant difference (p = 0.94), females predominated among myringosclerosis subjects (60.3% against 39.7%). If this trend were to be confirmed, a higher occurrence of myringosclerosis would be expected in group B, which contained many more women. Table 1, however, shows that the opposite was found, strengthening the hypothesis that CRF is a predisposing factor for middle ear calcification.

There was a statistically relevant difference between both groups relative to skin color. We do not believe, however, that this difference would have influenced the results of our comparison of the occurrence of myringosclerosis between groups A and B; there have been no reports indicating any statistically relevant difference in the prevalence of tympanosclerosis between races. A comparison of the frequency of myringosclerosis between the three main age groups revealed no significant difference, excluding the possibility that the former variable would have altered the results.

The age distribution was different between both groups. There is a controversy in the literature about whether any age range would show a higher prevalence of tympanosclerosis. Some authors have stated that this condition has a higher prevalence in children, since they are subject to frequent otitis media and surgery for placing ventilation tubes. Other believe that, as tympanosclerosis is an irreversible chronic process, it would be more prevalent in an older age range.^29^, ^32^ Møller,^10^ however, has demonstrated that this process may be reverted.

There appears to be an age-related bimodal curve in the prevalence of tympanosclerosis; a first peak occurs in children, who are at risk for otitis and ventilation tube surgery. A second peak occurs after age 40 years, when the progressive and irreversible nature of adult tympanosclerosis speaks louder. A lower prevalence zone between these two peaks might be explained by the spontaneous regression of this condition that is observed in children. Our sample does not allow an adequate assessment of this age group, since it did not include children or teenagers. The adults in our sample (groups A and B) had a higher occurrence in an intermediate age range (Charts 1, 2 and 3), between ages 21 and 40, and 41 and 60 years. There is, however, a frequency inversion in these two are ranges between both groups, where subjects aged over 40 years predominated in group A (Tables 2 and 3). This finding might have influenced the final result, although the paper we reviewed did not reach a consensus about the relation between tympanosclerosis and age. An evaluation of the occurrence of myringosclerosis in each of those two age ranges (Tables 4 and 5) reveals that the difference we found was smaller than the difference found in the global analysis (PR = 1.29 and p = 0.39 for ages 21 to 40 years, and PR = 1.37 and p = 0.11 for ages 41 to 60 years), which was, however, not statistically significant. We cannot state with certainty whether CRF contributed to the onset of myringosclerosis in our sample, although we repeatedly found a trend indicating a higher occurrence of myringosclerosis in CRF patients. It should be borne in mind that ageing tends to coincide with the presence and severity of renal disease; these become confounding variables in this type of study. Possibly an age-paired control group would clarify this matter.

There was no significant difference between group A subjects with and with no myringosclerosis relative to the duration of dialysis (p = 0.37). Kabaya et al.^33^ were also unable to find any influence from the duration of dialysis on the incidence of vascular calcification in hemodialyzed patients. Leskinen et al.^34^ also found no significant difference in carotid calcification in CRF patients before or after dialysis. Although various authors contend that metabolic changes induced by dialysis might operate on the etiology and pathogeny of ectopic calcification in CRF patients, there is no consensus in the literature on this subject. Our results suggest that the duration of hemodialysis does not appear to have a significant role in the onset of tympanosclerosis, event though this condition is possibly influenced by renal disease.

Serum calcium and phosphorus, measured in part of the CRF patient sample, were not different between patients with positive or negative otoscopies. Low or elevated calcium or phosphorus levels in these patients did not change the occurrence of myringosclerosis (p = 0.70 and p = 0.982) as shown on Tables 6 and 7. Table 8 shows that there was a higher occurrence of myringosclerosis in patients with normal serum PTH levels (22.7% compared to 10.8% in low serum PTH and 11.7% in elevated serum PTH levels); this finding, however, was not statistically significant (p = 0.192). At first sight, if there were a cause-effect relation between CRF and tympanosclerosis, the etiological and pathogenic mechanism of this condition would be similar to that found in vascular and visceral calcification, as Alfrey^20^ has reported. Serum calcium, phosphorus and PTH, however, were measured only once in this trial upon inclusion of patients; these measurement, therefore, are a picture of the metabolic situation at a given moment, rather than a well-established dysmetabolic pattern in these patients. Their calcium, phosphorus and PTH levels may vary significantly according to the progression of kidney failure, to dialysis and to other associated conditions such as diabetes, the diet and others.

The variables we studied suggest that CRF does have a significant influence on the onset of tympanosclerosis. The mechanism whereby renal disease generates tympanic sclerosis, however, is not clear from the data in this study, as the metabolic changes we studied and the duration of hemodialysis appeared to have no influence on the frequency of myringosclerosis. The literature states that the effects of altered calcium, phosphorus and PTH levels on pathological calcification may take place without any serum elevation of these factors being detected. It has been well defined that ultra-structural changes in tympanosclerosis are similar to those found in vascular and visceral calcification in CRF patients. Furthermore, as the serum metabolic changes in these patients are present systemically, it is logical to infer that their effect on the calcification process takes place similarly in the lamina propria of the middle ear mucosa. This is even more evident in the group of patients that presented myringosclerosis without an otological history of inflammation, where the only clear cause was renal disease, notwithstanding the aforementioned memory bias that might have affected these results to some uncertain degree.

Finally, if we look only at the control group, it could be stated that the prevalence of tympanosclerosis in persons aged over 20 years is 5%. It is possible that this percentage might have been even higher if we had included subjects aged 0 to 20 years; this, however, was not the aim of this study.

## CONCLUSION

There was no influence of serum levels of calcium, phosphorus or the PTH on the occurrence of myringosclerosis in the study group.

There was no influence of the duration of hemodialysis on the occurrence of myringosclerosis in the study group.

There was a statistically significant difference between the CRF patient group and the control group in the frequency of myringosclerosis.

These data, however, cannot confirm with certainty that CRF had any relation with the increased occurrence of myringosclerosis, as the variable age might have influenced the results.
